# Shapes of minimal-energy DNA ropes condensed in confinement

**DOI:** 10.1038/srep29012

**Published:** 2016-07-01

**Authors:** Antonio Šiber

**Affiliations:** 1 Center of Excellence for Advanced Materials and Sensing Devices, Institute of Physics, Bijenička cesta 46, 10000 Zagreb, Croatia

## Abstract

Shapes of a single, long DNA molecule condensed in a confinement of a virus capsid are described as conformations optimizing a model free energy functional accounting for the interplay between the bending energy of the DNA and the surface energy of the DNA bundled in a “rope”. The rope is formed by bundled DNA brought together by (self-)attractive interactions. The conformations predicted by the model depend on the shape of the confinement, the total amount of the packed DNA but also on the relative contributions of the bending and surface energies. Some of the conformations found were not predicted previously, but many previously proposed DNA conformations, some of which are seemingly contradictory, were found as the solutions of the model. The results show that there are many possible packing conformations of the DNA and that the one which realizes in a particular virus depends on the capsid geometry and the nature of condensing agents.

Organisms have developed mechanisms to physically compress the DNA strand(s) in order to fit in the crowded space of the cell - the use of proteins to condense the DNA in chromosomes^1^ is typical both in the eukaryotic and prokaryotic cells, but also in viruses which sometimes use specific proteins to confine their DNA in a capsid (e.g. adenoviruses, see refs 2 and 3). The DNA molecule carries a large negative charge, so that its confinement requires screening agents^4^ - that is why the “condensing” proteins typically have a pronounced positive charge.

The role of screening of (double stranded) DNA self-interaction is in some bacteriophages, e.g. T4, played also by multivalent counterions, such as polyamines found in bacteria where bacteriophages assemble^5^. Multivalent counterions are known to induce *condensation* of DNA, i.e. the effective self-attraction of the DNA^6^. The viral DNA is thus expected to be condensed in cases when the virus capsid is impermeable to condensing counterions, so that they, once packed in the bacteria/cell, cannot leave the virus once it enters the inter-cellular space. The role of the condensing agents may be very different in the process of DNA packing^7^,^8^ and in the condensation of the DNA once it enters the virus - the later is of primary interest to this work.

Shapes of many virus capsids are experimentally determined to high precision, but the conformation of DNA in viruses (bacteriophages in particular) is less well known and many different shapes and orderings of the viral DNA have been proposed. Condensation of DNA in free space has been investigated *in vitro*. The shape of the DNA condensate is typically toroidal^9^. In such a configuration a single, long DNA strand is wound in a toroidal “rope” so that it forms locally parallel DNA streamlines (“bundle” of DNA) enabling thus the DNA self-attraction. The shapes and sizes of the DNA toroids have been theoretically explained^10^ in a model of the condensate that accounts for the costs of the DNA bending and the formation of the condensate surface. In order to apply the model to viruses one needs to consider the DNA condensation in the confinement of the capsid. Such calculations have been performed^11^,^12^, but the toroidal symmetry of the condensate was *assumed* from the start. The obtained theoretical predictions were found to be adequate for T5 bacteriophages which were experimentally modified so to eject about 60% of their DNA with the remaining part condensed using a buffer containing spermidine or PEG. A question still unresolved is^13^ whether the *fully packed* bacteriophages and other dsDNA viruses (e.g. herpes) contain the condensate with toroidal (axial), the so-called *inverse spool* symmetry, and whether such conformation is universal. Non-toroidal conformations of bundled DNA have been proposed previously, most notably by Hud^14^, Petrov *et al*.^15^ and Earnshaw *et al*.^16^ (for the partially disrupted capsid of giant T4 bacteriophage). Hud proposed a conformation where the DNA is packaged in a toroid that is “folded” forming thus a highly compact structure. Similar conformations may have been detected in numerical simulations^15^,^17^,^18^. However, the energetic, dynamic and possibly entropic reasons for the formation of the “folded toroid” configuration are still not obvious and additional investigation is warranted. Furthermore, it is not clear whether the folded toroid conformation is somehow special or it may be only a member of a set of possible conformations of a circularized DNA bundle, favorable energetically or dynamically in given circumstances. In this respect, it is important to investigate the interplay of surface and bending energies and how their competition influences the formation of the confined DNA structure - this it the aim of the present paper. Brownian dynamics simulations of the DNA packing^15^ revealed that the DNA conformation may depend on the shape of the virus capsid, i.e. whether it is elongated or not. The influence of the geometry of the confinement on the shape adopted by the DNA condensate is thus of interest and will also be investigated here.

Previous investigaions indicate that a unique DNA conformation is not likely to be representative for all the DNA phages, irrespectively of the capsid geometry (which may be quite elongated in some bacteriophages and can have large internal portal structures^19^) and of the condensing agent (dispersed proteins, proteins bound to capsid, or multivalent counterions)^20^ - the present study aims to clarify all of this factors and their influence on the conformation assumed by the condensed DNA.

The DNA conformations are investigated here within a framework of the model used to explain formation of DNA toroids in free space^10^. The shape of the DNA condensate is thus assumed to minimize the free energy but the model in ref. 10 is extended suitably to allow for investigation of non-toroidal conformations of condensed DNA. Such an approach is advantageous with respect to the simplicity of modeling as it requires only two parameters for competing energies: the persistence length (i.e. the bending rigidity) of DNA, *L*_*p*_, and the surface energy per unit area of the DNA rope, *σ*. The shapes of interest can be most easily visualized as solids generated by motion of a planar domain 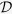 of area *S* bounded by curve 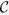 (cross-section of the bundle) along a *closed* three-dimensional curve Γ of length 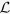 so that the geometric centroid of 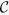 lies on Γ. This produces a shape (rope) of volume 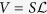 and area 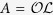 where 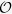 is the length of 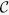. Physically, the rope is realized by DNA wounding many times around Γ, forming locally a bundle of parallel DNA streamlines. The curve Γ needs thus to be closed, which makes the problem importantly different from and more difficult than those investigated in the previous studies of shapes of open strings in confinement^17^.

The DNA ropes contain the bundled DNA, together with condensing agents. It is easy to conceptualize the distribution of small multivalent couterions in a DNA rope, but the model in principle applies to condensing proteins also - in this case, the proteins may disturb the DNA streamlines around them, but the notion of the rope may still make sense. For example, when the capsid of adenovirus is disrupted, the DNA is detected in a form of a thick fiber, most likely containing several DNA strands (“string”) complexed with condensing proteins (“bead”)^2^, so that the notion of a “nucleoprotein filament”^21^ (the rope) within a capsid may make sense.

Concerning the model, one could allow for the variation of cross-section bounding curve along Γ, as long as its area remains the same - this may simulate rearrangements of DNA strands in a cross-section, depending on its position on Γ. Such rearrangements may be required for tighter packing of DNA in the capsid. In the model adopted here, the cross-section of the bundle is assumed to be a circle of radius *r*, for all points on Γ. This simplifies the analysis, as for given *r*, one needs to determine only the optimal path Γ. Note, however, that for the given *volume* (or length) of packed DNA, the shapes differ depending on *r* - larger cross-sectional areas imply shorter paths Γ, as *V* is preserved. DNA ropes dominated by surface energy (*σA*, where *σ* is the DNA surface tension) are expected to be stout and compact, i.e. their cross-sectional areas should be as large as possible so that their exposed surface is minimal. On the other hand, ropes dominated by elastic energy are expected to be slim and long so to maximally reduce the curvatures of individual DNA strands - this implies smaller cross-sectional areas^10^.

The total (free) energy of the condensate in the described approach is


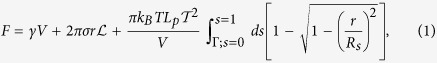


where the first term represents condensation energy of the rope (*γ* is the condensation energy per unit volume and may be positive if the DNA-DNA separation, *d*, is smaller than the optimal), the second is the surface free energy (including also the entropic contribution of the DNA strands at the bundle surface), and the third is the bending energy. The model does not include the DNA twist explicitly. As in ref. 10, the surface energy is assumed to be proportional to the exposed surface of the bundle, 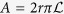, with the surface tension, *σ* as the proportionality coefficient i.e. the energy scale. The bending energy is obtained by integrating the squared curvature over the whole length of DNA, 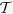. This can be reduced to the single integral once the integration over the circular cross-section of the rope/bundle is performed. The remaining integral in Eq. 1 is performed over the centroid curve Γ, where *s* is the arc-length coordinate of Γ (*s* ∈ [0, 1]), and *R*_*s*_ is the radius of curvature at the center of the circular cross-section. Bulk and surface energy parameters, *γ* and *σ*, depend on *d* and are, in the simplest approximation, proportional^22^.

For known energy parameters *σ* and *L*_*p*_, and fixed DNA length (or volume - these are related through *d*^22^) of DNA, the minimization of *F* reduces to finding optimal *r* and Γ which are mutually dependent as the total DNA volume, 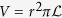, is fixed. The minimization of *F* with respect to centroid curve Γ and radius *r* needs to account for (*i*) the confinement and (*ii*) possible self-collisions of the bundle in cases when the curve Γ approaches itself. Both physical effects are essentially important for the problem in question and both can be treated as penalizations of the energy functional in Eq. (1). It should be noted that in the limit of a thin bundle the approach enables one to follow essentially a *single* DNA strand - in this limit it is thus complementary to numerical simulations of packed DNA^8^,^15^ and other models of confined semiflexible polymers^23^.

For given cross-sectional radius *r*, infinitely many closed curved Γ can be constructed, and finding the one which minimizes the free energy is the aim of the numerical procedure described in Methods. The minimization is performed somewhat asymmetrically, due to the fact that the surface energy depends only on *r* and not on Γ. For given volume *V*, specification of *r* uniquely determines the surface (free) energy, and the minimization of *total* free energy reduces to finding Γ which, for given *r*, yields minimal bending energy of the rope. The optimal shape of the rope depends on the magnitudes of elastic and surface energy parameters, *L*_*p*_ and *σ*, respectively. However, whatever their magnitudes may be, the rope will eventually have certain cross-sectional radius *r* and centroid curve Γ. Instead of considering three-parameter space (*V*, *L*_*p*_, *σ*), it suffices to consider two-parameter space (*V*, *r*), as the rope radius “encodes” the relative importance of surface vs. bending energy of the rope. There is a bonus to this representation, because it should enable a simpler comparison between the theory and experiments - whereas the bending and surface energy parameters are inaccessible from the experiments, the rope radius should not be. The problem is thus reduced to *geometry*. For given *V* and *r*, Γ is found such that the bending energy *only* is minimal. The rope radius *r* (and the corresponding surface free energy) is systematically modified and the minimization of bending energy performed separately for each value of *r*. Note that in this representation the results do not depend on the value of persistence length *L*_*p*_, as it appears only as the scale (multiplying factor) of the bending energy. One should remember, however, that small persistence lengths diminish the contribution of the bending energy in the total free energy balance, emphasizing importance of the surface energy contribution - in such case, one may expect formation of ropes with large values of cross-sectional radius^10^.

The bacteriophage capsids are either icosahedral or elongated along an axis connecting two opposite icosahedron vertices (prolate), such as e.g. T4^16^,^24^. In the calculations, the confinement (capsid) is represented by a cylinder of base radius *r*_*B*_ and height 2*r*_*B *_*f*, where *f* is the “elongation factor” - *f* = 1 approximately represents nearly isometric virus particles, and *f* > 1 prolate viruses. The two-parameter space (*V*, *r*) is appropriately scaled so that the two relevant parameters are ratio of radii, *p*_1_ = *r*/*r*_*B*_ (*p*_1_ < 0.5), and the volume ratio, 
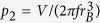
. In a wild-type virus, volume ratio is determined by the length of the viral genome. It may be useful to consider it as a parameter in the minimization as such calculations may give insight in the structures of DNA condensates in partially filled viruses, possibly also during the packaging or ejection process^25^, although one should always keep in mind that the structures obtained here are thermodynamical minima and may not be realized dynamically^26^. For a certain volume of the DNA (fixed *p*_2_) there are solutions with different bundle radii (*p*_1_). For given *σ* and *L*_*p*_, only one of these radii minimizes the total free energy. The values of *σ* and *L*_*p*_ depend on the ionic content of the bacteriophage interior and presence of the condensing proteins.

## Results

**Figure 1:**
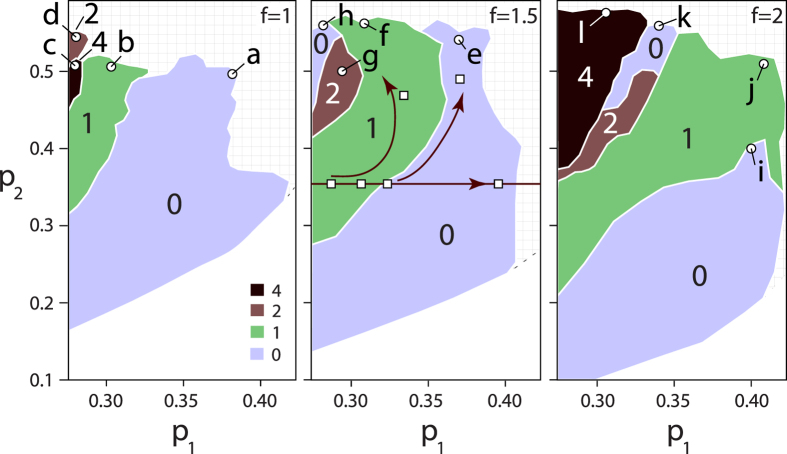
Regions of (*p*_1_, *p*_2_) space with different absolute values of writhe as indicated. In white regions the shapes are not influenced by the confinement - the (non-selfintersecting) solutions there are tori. In cross-hatched regions, the solutions were not found - all the shapes in these regions either penetrate the confinement or self-intersect. Shapes denoted by circles (squares) are represented in Fig. 2 (Fig. 3).

The applicability of the model to viruses depends on the range of parameters examined. If the model is understood in its most restricted sense - to represent the condensed DNA “rope” - the ratio of radii needs to be sufficiently large. This is due to the *continuum* concepts of bulk and surface DNA in the model, so that the number of DNA streamlines in the cross-section 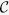 needs to be large enough in order that the two concepts make sense. In the condensed DNA medium, the center-to-center distance between the DNA strands/streamlines is about 3 nm^9^,^12^. Assuming a hexagonally packed DNA^12^, the notion of “bulk” and “surface” may make some sense for at least 7 DNA helices, close-packed so that the central helix is surrounded with six equally distanced neighbors. The effective radius of such a bundle would be about 4 nm (3 + 1.5 nm). The size of the capsid depends on the bacteriophage type, but taking, e.g. *ϕ* 29^25^ with a base radius of about 25 nm, the minimum ratio of radii that could be simulated by the model would be about *p*_1_ = 4.5/25 ~ 0.2. For larger bacteriophages, such as T4^24^, the minimum ratio is about *p*_1_ = 4.5/45 ~ 0.1. In the simulations presented below (Fig. 1), *p*_1_ > 0.275. As demonstrated, smaller values of *p*_1_ (~0.1) may also be of interest - these shapes with small cross-sectional radii occur for sufficiently low surface tensions. The numerical method adopted here, however, becomes increasingly slower as *p*_1_ is diminished. This is due to the fact that, for given volume *V*, slim ropes (small *p*_1_) are longer, requiring effectively more degrees of freedom for description of centroid curve Γ (see Methods). This increases the number of configurations which need to be examined by the minimization procedure.

The minimal energy shapes can, of course, be plotted once found, yet a quantitative measure is required to classify them. An adequate measure was found to be the *writhe* of Γ (Wr) which quantifies the topological complexness of the rope - in two dimensions, writhe is the total number of self-crossings of Γ, which, in general, contains both positive and negative contributions, depending on whether the curve crosses itself from “above” or “below”^27^. In three dimensions (3D), the writhe can be evaluated by performing a double integral over the closed curve^27^. For our purposes it suffices to consider the absolute value of writhe, |Wr|, as shapes with the same absolute value of Wr have the same free energies. Writhes obtained in 3D are real numbers, yet the shapes are found to belong to *classes* represented by territories in parameter space, in which their writhe is typically within ~0.25 around the integer value - there are some exceptions, specified below. Writhes of minimal free energy shapes in a (*p*_1_, *p*_2_) parameter space are shown in Fig. 1 for elongation factors *f* = 1, 1.5, 2 (from left to right). The elongation factors chosen can be compared to real bacteriophages. Elongation factor *f* = 1 roughly corresponds to bacteriophages with isometric icosahedral capsid, such as T12 and *λ* phages. T4 phage has an elongated icosahedral capsid whose height to width ratio is about 1.35^24^, similar to *ϕ* 29 (1.35^25^) and T2 (1.45^14^) bacteriophages. In giant vibriophages^28^, the elongation ratios are between 1.7 and 2.1. Values of *f* = 1.5 and *f* = 2 are thus relevant to real bacteriophages. Much larger elongation ratios are found in giant headed T4 phages^16^ which have a variable head morphology (elongation ratios from ~2.5 to ~12) due to a mutated coat protein.

**Figure 2:**
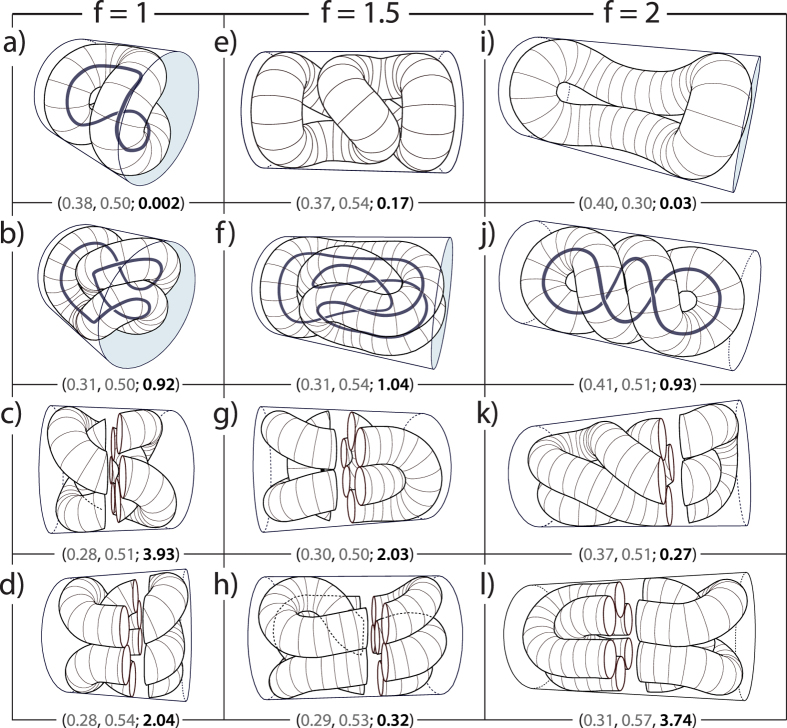
Minimum free energy shapes for *f* = 1, *f* = 1.5, and *f* = 2 (left, middle, and right columns). Below each of the shapes displayed, the corresponding *p*_1_, *p*_2_ parameters and the writhe are indicated as (*p*_1_, *p*_2_; Wr). To better represent the three-dimensional nature of the shape, in panels a,b,f,j, the centroid curves of the shapes, Γ’s, are shown. In panels c,d,g,h,k,l cylindrical slab is cut out, so that the cross-sections of the shapes, parallel to the confinement cylinder bases can be seen.

The territories with different writhes have sharply defined borders so that the solutions from the two sides of the border differ by |ΔWr| = 1 or more. The exceptions were found for *f* = 1.5 in neighboring regions having Wr = 0 and 2, and for *f* = 2 in neighboring regions Wr = 0 and 1 and Wr = 2 and 0 - there the transitions between different conformations are continuous and the borders are fuzzy. A particularly densely packed shapes (large *p*_2_ parameters) with different writhes were selected for graphical representation. These are indicated by circles and letters which correspond to panel labels in Fig. 2.

In case of isometric capsids (*f* = 1), a large portion of the parameter space is occupied by shapes with Wr = 0. These shapes are “squeezed” and folded tori, which become maximally folded for large packing ratios *p*_2_, as shown in Fig. 2a (*f* = 1). Exactly such shapes were predicted for organization of double-stranded DNA in the bacteriophage heads by Hud^14^ (see Fig. 2b in ref. 14). The results obtained here show that the folded toroid proposed by Hud is the shape with minimum free energy for sufficiently thick DNA ropes/condensates (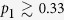). The maximum packing fractions that can be obtained with such a shape are about *p*_2_ ~ 0.5. As also explained by Hud^14^, further increase in the packing fraction would be possible by allowing for a change in the cross-section of the DNA condensate along the centroid curve. Intriguingly, Hud’s shape seems to be quite generic as it appears also in different contexts and has been found in simulations of growth of thin, frictionless and non-attracting elastic filaments in spherical confinement^17^,^18^.

Shapes with W*r* = 1 in isometric case can be conceptualized as an additional coil turn of the DNA rope sandwiched between the folded toroid (Fig. 2b, *f* = 1). For *f* = 1, this packing pattern produces a maximum packing fraction of about *p*_2_ = 0.51, but more efficient packings with the same overall pattern are possible in case of elongated capsids (*p*_2_ = 0.54), as shown in Fig. 2f for *f* = 1.5. The shapes could also be understood as pieces of tightly packed helix found in open tubes^17^, but in this case their appearance is complicated by the requirement on Γ curve being closed. A surprising conformation was found in a narrow range of parameters for isometric capsids where W*r* ≈ 4 (Fig. 2c, *f* = 1). The conformation of the DNA rope there is *knotted*, which can be most easily detected by removing the confinement once the minimal shape is found and letting it relax - a prototypical trefoil knot appears, with three-fold symmetry and maximally extended loops so that their curvature is minimal. The writhe of the relaxed shape drops by one, to ≈3. The knotted shapes cannot thus be unfolded to a toroid once the capsid is destroyed - this is a situation quite different from the one discussed by Hud^14^ and applies also to the shape shown in Fig. 2l, representative for the knotted shapes in the large region of parameter space when *f* = 2 (see below).

In addition to reproducing Hud’s shape, the calculations also reproduce the “supertwisted” shapes proposed by Earnshaw *et al*. for partially disrupted capsids of giant T4 phages^16^ (the “supertwisting” of a thick DNA rope found here should not be mistaken for supercoiling of a *single* DNA strand - see e.g. ref. 29). The shape which Earnshaw *et al*. describe as “twisted skeins of yarn” is quite similar to the one found here (twice twisted closed loop - “double helix”) for minimal energy condensates in *f* = 2 capsids having Wr ≃ 1 (Fig. 2j). The elongation ratio of capsids containing supertwisted DNA in experiments^16^ is about *f* ~ 4. A similar shape, but twisted only once, has been found by Petrov *et al*., also in elongated capsids^15^. The model elaborated here does not explicitly include the twisting contribution to elastic energy of the condensed DNA rope. The DNA twist may be important for the condensation, especially for its dynamics, as shown e.g. in ref. 30. However, it is interesting to note in this context that the DNA twist is *not* required to obtain some of the rope conformations whose shape might suggest otherwise. For example, the appearance of a shape similar to the one shown in Fig. 2j (but twisted only once) has been previously proposed to result from the twisting contribution to the DNA elastic energy^31^. In the model presented here, such a shape occurs due to a particular interplay of the DNA rope bending energy and the packing constraints effected by the rope confinement in the cylinder and its self-collisions.

In many densely packed conformations of bundles in elongated cylinders, DNA strands are mostly parallel to the cylinder axis (see Fig. 2f,h,k,l), a feature found also by Earnshaw *et al*. in their experiments^16^ which led them to propose the model of coiled DNA with the axis of coiling perpendicular to the elongated axis. Some of the structures found could also be loosely interpreted as a *concentric* spool^15^ (“ball of yarn”), without axial symmetry, see e.g. Fig. 2c - there the DNA strands spool along different axes, leaving a hole in the middle of the capsid. When viewed from a fixed perspective many of the shapes would appear as seemingly “random” configurations of short, liquid-crystalline like segments of DNA - this is due to complicated connectivity of the shapes (see e.g. Fig. 2c,h) and the large part of their volume being obscured from view. In that respect, some of the shapes obtained are consistent with the “liquid crystalline drop” model^13^,^32^ and the cross-sectional size of the hexagonally crystallized domains of DNA in that model corresponds to the rope diameter 2*r*. The message here is that different models of DNA packing in bacteriophages proposed previously can be reproduced in the present model for different values of *p*_2_ and *p*_1_, i.e. depending on the total length of the DNA and relative importance of surface vs. bending energy of the DNA condensate - this depends on the chemical environment in a capsid which determines the bulk and surface energy of the DNA bundle but also influences the persistence length of DNA. The predictions of model are, however, not consistent with the previously proposed conformations which require sharp bends of the DNA, such as the so-called spiral fold model proposed by Black *et al*.^33^.

**Figure 3:**
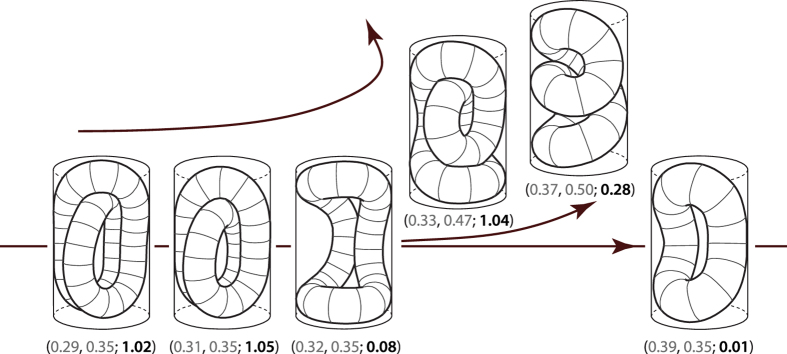
Minimum free energy shapes for *f* = 1.5 denoted by squares in Fig. 1. Below each of the shapes displayed, the corresponding *p*_1_, *p*_2_ parameters and the writhe are indicated as (*p*_1_, *p*_2_; Wr). The arrows suggest the path through the (*p*_1_, *p*_2_) space, all up to shapes denoted by f and e in Fig. 1 in Wr = 1 and Wr = 0 regions, respectively.

Figure 3 illustrates how the conformations of the minimal energy ropes vary throughout the (*p*_1_, *p*_2_) space. The shapes chosen for representation are denoted by squares in Fig. 1 for *f* = 1.5. The figure illustrates how the shapes with the same volume ratio (*p*_2_ = 0.35) change as the rope radius is increased (from left to right; first, second, third and sixth shape). Note how the conformation of the rope changes as the border between the two writhe region is crossed (between the second and the third shape, the writhe changes from ~1 to ~0). The two additional shapes displayed (for *p*_2_ = 0.47 and 0.50) enable one to visualize how the shapes vary in the Wr = 1 and Wr = 0 regions, all until the shapes denoted by f and e in Fig. 1 are reached, as indicated by arrows in Figs 1 and 3.

**Figure 4:**
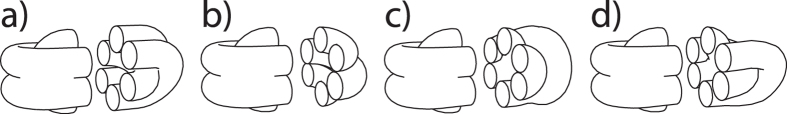
Four different closures of the three-toroids motif resulting in shapes having a single, closed centroid curve.

A specific *motif* can be detected in both knotted and unknotted shapes, such as those shown in Fig. 2d,f,g. Roughly, the motif consist of three half-tori, two of them lying in parallel planes and placed one above the other, and the third one situated in the void created by such an arrangement, lying in a plane perpendicular to those of the two parallel half-tori - an efficient packing of such sort is possible when the major radii of the tori are about two times larger than the tube radius *r* (*p*_1_ ~ 0.3). Often, the two parallel half-tori are found to be slightly folded, upwards and downwards for a better packing (see left parts of the shapes in Fig. 2d,g, and right part of the shape in Fig. 2l). Some of the shapes with large packing fractions can be understood different closures of the three-toroid motif, as illustrated in Fig. 4. A closure of the motif shown in Fig. 4a corresponds to minimal energy shapes in Fig. 2c,l - this case of closure is particularly interesting as the conformation of the closing shape is the same as the motif it closes, only rotated by ~60 degrees around the cylinder axis. Closures in Fig. 4b–d correspond to minimal energy shapes in Fig. 2d,f,g, respectively. All of the shapes with the characteristic motif shown have large packing fractions, *p*_2_ > 0.5.

A finite thickness of the confined DNA rope is what makes the appearance of certain conformations possible only in some regions of parameter space. For example, the minimal length of the rope required to tie a trefoil (3_1_) knot depends on the rope radius, so that 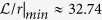
34 - the trefoil knots formed by ropes with ratios 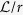 larger than this (loose knots) can be tied, while those with smaller can not. Combining parameters, one finds that 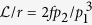. The trefoil knot is the simplest of all the knots and the 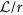 ratio (rope length) required to tie it is the smallest^34^. The next shortest length knot is 4_1_ knot, requiring 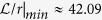, but as the rope length increases, the space of knots that can be tied rapidly expands. All of these knots are candidates for the solutions of the problem, and are interesting in particular when stiffly tied (densely packed), i.e. as soon as the rope length 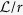 becomes larger than a critical value (32.74 and 42.09 for 3_1_ and 4_1_ knots, respectively). The trefoil knots were found by the SA procedure starting from random and torus conformations, so no specific initial configurations were required, yet one may wonder whether more complicated knots may evade detection. To investigate this, knots whose coordinates were taken from ref. 34, with simply scaled centroid curves so to account for ratios 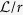 larger than the minimal, were used as initial conformations in the SA procedure with different initial temperatures. More complicated knots were not found to be the minimal energy solutions for the *f* factors investigated when *p*_1_ > 0.275. One should also note that the knotted conformation when *f* = 1 appears for rope lengths significantly larger (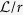 ~ 50) than in the case of tight knot, so that the centroid curve of the observed conformation is significantly deformed with respect to the centroid curve of the tight knot in order to fit in the cylinder (see Fig. 2c).

## Discussion

All the findings indicate that there may not be a unique model of the DNA conformation appropriate for all bacteriophages. The conformation with minimal free energy depends sensitively on the total amount of the DNA, the nature of the condensing agents, and the shape of the bacteriophage capsid. The phase space of conformations is divided in territories of different writhe, separated mostly by sharp borders, which do not allow for a continuous change of the conformation. Some of the conformations proposed previously were also found in the present work, in different positions in the parameter space, further corroborating the idea of the non-uniqueness of the DNA packing in bacteriophages. The results indicate that various conformations proposed previously may be understood within the same theoretical framework, as minimal-free-energy states of the DNA condensate, obtained in particular condensation conditions, for given amount of the DNA and in a geometrical confinement provided by the shape of the capsid. They thus interlink the previous studies (e.g. Hud’s folded toroid^14^, Earnshaw’s *et al*. twisted skeins of yarn^16^ and Lepault’s *et al*. liquid crystalline drop^13^,^32^), showing that different shapes of confined DNA condensates may be understood in a unifying model.

## Methods

The DNA rope was represented by a string of intersecting spheres, all with the same radius *r*, with centers distributed on centroid curve Γ and separated by a fixed amount *qr*, *q* ~ 1^35^. The total volume of such a system is proportional to the number of spheres, *N*. The degrees of freedom of the rope are related to the positions of sphere centers on Γ. The self-collisions of such a shape are easily checked for by calculating the distances between all the spheres. Once detected, the energy functional are penalized to essentially forbid the sphere-sphere penetration for spheres that are not nearest neighbors along Γ. Collisions of the shape with the container (capsid) are detected by calculating the distances between all the spheres and the cylinder surface (including cylinder bases) and by penalizing the functional to forbid the penetration of the rope outside the container. The closedness of Γ is imposed by penalizing the energy functional when the distances between the first and the last (*N*-th) sphere are larger or smaller than *qr*. The elastic energy is calculated from the positions of all the spheres by interpolating a cubic spline through all the points and integrating its curvature along Γ, as specified in Eq. (1). The minima of free energy functional, penalized as explained, are found by the simulating annealing procedure. The numerical procedure was run many times, with different initial conditions (i.e. initial shapes at high temperatures of the algorithm) for Γ, including random curves, circles, spirals/coils, (trefoil) knots, and appropriately modified curves found by the algorithm for values of *p*_1_ and *p*_2_ parameters different from the one considered in the minimization. The solutions were carefully manually sifted, some of them reselected for repeated minimization, all until no better solutions could be found.

## Additional Information

**How to cite this article**: Šiber, A. Shapes of minimal-energy DNA ropes condensed in confinement. *Sci. Rep.* **6**, 29012; doi: 10.1038/srep29012 (2016).
